# Effects of *Artemisia annua* on experimentally induced leucocytozoonosis in chickens

**DOI:** 10.1016/j.psj.2021.101690

**Published:** 2021-12-30

**Authors:** Yu-Huan Chiang, Yen-Cheng Lin, Sheng-Yang Wang, Yen-Pai Lee, Chih-Feng Chen

**Affiliations:** ⁎ Department of Animal Science, National Chung Hsing University, Taichung 402, Taiwan; † Department of Forestry, National Chung Hsing University, Taichung 402, Taiwan; ‡ Agricultural Biotechnology Research Center, Academia Sinica, Taipei 115, Taiwan; § The iEGG and Animal Biotechnology Center, National Chung Hsing University, Taichung 402, Taiwan

**Keywords:** *Leucocytozoon caulleryi*, leucocytozoonosis, *Artemisia annua*, *Culicoides arakawae*, molecular diagnosis

## Abstract

The biting midge *Culicoides arakawae* is the vector for the parasite *Leucocytozoon caulleryi*. Birds infected with *L. caulleryi* develop leucocytozoonosis. Given the food safety concern regarding drug residue in eggs, discovering a natural alternative to antibiotics is a worthy of exploration. Thus, we investigated the effects of the antimalarial herb *Artemisia annua* on experimentally induced leucocytozoonosis in chickens. We reared *C. arakawae* in the laboratory. Eggs were cultured, developing into larvae, pupae, and imagoes. Female midges sucked the blood of sick chickens and then were ground into a solution injected into healthy chickens. The control group was given empty capsules daily, whereas the 2 experimental groups were given 40 mg/kg sulfadimethoxine or 0.5 g of *A. annua* powder. *Leucocytozoon* gametocytes were detected in chicken blood through Giemsa staining. PCR detected the *cytochrome b* gene of *L. caulleryi* in the infected chickens. No significant among-group differences in body weight gain were observed before d 14 postinoculation (*P* > 0.05). Body weight gain in the control group was significantly lower from day 14 to 28 postinoculation (*P* < 0.05). After day 14, rectal temperature in the experimental groups decreased significantly compared with that in the control group. Lower rates of pale comb and green feces were observed in the animals receiving treatment from day 0. The experimental groups had a higher recovery rate and recovered earlier than did the control group. By day 31, all the animals had recovered. PCR detected *L. caulleryi* in the infected chickens with high sensitivity and accuracy. The animals receiving *A. annua* exhibited increased weight gain and reduced parasite concentrations in the blood. This in turn reduced mortality and the occurrence of pale comb and green feces. The findings are informative for research on leucocytozoonosis.

## INTRODUCTION

Leucocytozoonosis is a parasitic disease affecting birds that is relatively common in Asia and is caused by infection with *Leucocytozoon caulleryi*. Outbreaks typically occur in summer. In chickens, leucocytozoonosis cause anemia, hemorrhage-induced death, reduced egg production in laying hens, and retarded growth in chicks. Mathis and Leger (1909) first detected *L. caulleryi* in the blood of domestic fowl in the Tonkin region of Vietnam. Since then, relevant evidence from Malaysia (Kuppuswany, 1936), Japan (Akiba et al., 1958), Myanmar (Win et al., 2020), and Taiwan (Lee et al., 1966) has been presented. Through further investigation into leucocytozoonosis, Akiba (1960) discovered that the vector for *L. caulleryi* is a biting midge of the genus *Culicoides* specifically, *Culicoides arakawae*. The same author devised a laboratory method for the laboratory colonization of this species in an experimental environment resembling a paddy field. The results obtained let to the successful maintenance of the etiological agent of leucocytozoonosis in chickens (Morii and Kitaoka, 1968b). We previously improved upon this colonization method by using a chamber with a constant temperature of 25 ± 2°C, using an agar medium and feeding larvae with the soil nematode *Rhabditis* sp. The life cycle was completed in 18 to 26 day (Chen, 1989; Lien and Peng, 1989). On the basis of these findings, we experimentally infected chickens according to the regeneration time of *L. caulleryi*.

In the life cycle of *L. caulleryi*, a female *C. arakawae* transmits sporozoites to a healthy chicken. The sporozoite travels through the blood vessels to different organs, where it reproduces asexually, producing thousands of merozoites. The merozoites then enter red blood cells, where they develop into gametocytes (Morii et al., 1981; Chen, 1989). Gametocytes are ingested by a female midge when it feeds (by sucking the blood of the chicken). Next, these gametocytes mate in the gut of the gut of the midge and begin a cycle of growth and multiplication. After 2 to 3 day, the sporozoites migrate to the salivary glands of the midge. When the midge bites another chicken, anticoagulant saliva is injected together with the sporozoites, which migrate to the organs and thereby begins a new cycle.

Conventional methods for diagnosing leucocytozoonosis, mainly involve the clinical symptoms, the examination of chickens’ peripheral blood smears, and histopathological findings in organs. These diagnoses largely depend on the protozoan life cycle. Notably, a new method provides the possibility of the sequence-based identification of different mitochondrial lineages within parasite genera (Hellgren et al., 2004).

Laying hens may be administered drugs such as antibiotics to prevent leucocytozoonosis, among other diseases. However, drug residue in eggs constitutes a serious food safety concern. Therefore, finding a natural alternative to antibiotics is a worthy undertaking. The plant *Artemisia annua*, known for its antimalarial properties, has been studied for its numerous biological activities. Artemisinin was one of the main active ingredients of *A. annua*. In a study on severe falciparum malaria, recipients of artesunate had a significantly lower mortality rate than recipients of quinine (14.7 vs. 22.4%; Dondorp et al., 2005). Moreover, *A. annua* has been determined to be effective in treating fever and mediating immunity. It also has demonstrated anti-inflammatory, analgesic, anti-schistosomiasis, antibacterial, and antiviral effects (Noori et al., 2004; Antoine et al., 2014; Wang et al., 2015; Tu, 2016).

In the first part of the study, we reared *C. arakawae* in a laboratory setting and established an animal model of leucocytozoonosis by experimentally infecting *L. caulleryi*. In the second part, we subjected the chickens’ blood and tissue samples to PCR for an early diagnosis of leucocytozoonosis, and explored the effects of *A. annua* on this disease.

## MATERIALS AND METHODS

### Laboratory Colonization of *C. arakawae*

Wild *C. arakawae* was captured from a chicken farm from 5 to 8 p.m. on April to October, 2019, using 0.25 mm x 0.25 mm mesh bag with a length of 68 cm and a width of 48 cm. After the midges were transported to the laboratory, they were reared in paper cups and fed with 10% sugar water in a chamber where the temperature and relative humidity at 25 ± 2°C and 90 ± 3%, respectively. Three days later, the female *C. arakawae* were moved into tubes with a 4 cm height and 0.5 cm width to spawn for 2 days. Thereafter, the eggs were placed, cultured, and maintained on 0.6% agar medium containing 5% dried yeast powder, 2% agar, and water. The larvae were fed with *Rhabditis sp*. A small pinch of the nematode was added to the larval medium daily. After the larvae developed into pupae, we separated the pupae from the agar medium and moved them into petri dishes. It took 3 days for the pupae to emerge imagoes. The female midges were allowed to feed on chicken 3 to 4 days emergence. Subsequently, the female midges were reared in paper cups for approximately 3 days until the eggs had matured.

### Experimental Infection of Chickens With *L. caulleryi*

Sick chickens, identified as those exhibiting symptoms of pale comb and green feces, were isolated from a laying farm. Gametocytes were examined through the Giemsa stain of blood smears. To regenerate *L. caulleryi* in the laboratory, in the 2 to 3 day after the emergence of the *C. arakawae*, the female midges were made to suck the blood of the sick chickens. Subsequently, these midges were reared in paper cups and fed with 10% sugar water in the chamber where the temperature and relative humidity were maintained at 25 ± 2°C and 90 ± 3%, respectively. Before experimental infection, the gravid female was dissected and confirmed that sporozoites had formed in salivary glands. Next, whole female midges were grated and filtered into the saline solution. After dilution, we injected sporozoites into healthy chickens through the wing vein.

### Diagnostic Methods

#### Blood Smears

Chicken blood was collected through the wing vein. We added 0.2 M ethylenediamine tetraacetic acid to the samples as an anticoagulant. The blood smears were fixed with absolute ethanol for 5 min and then stained with Giemsa's solution (pH 7) for 30 min. Almost the entire area of each of the stained slides was examined for *Leucocytozoon* gametocytes under a microscope at 200 × and 400 × magnification. The concentration of parasites in the blood was calculated by obtaining the percentage proportion of parasites to red blood cells in the blood.

#### Molecular Diagnosis

To detect *L. caulleryi* in the chicken blood, we amplified *cytochrome b* and *cytochrome c oxidase subunit 3* fragments with 2 pairs of primers designed on the basis of the mitochondrial DNA sequence of *L. sabrazesi* (Zhao et al., 2016). Through the use of the Biokit Blood or Tissue and Cell Genomic DNA Purification Kit (Biokit, Taiwan), DNA samples were extracted from the blood of the chickens and from their liver, pancreas, kidney, lung, spleen, myocardium, pectoral muscles, and leg muscles. PCR was conducted using a total volume of 10 μL containing 0.5 μM of each forward and reverse primer, 30 to 50 ng of genomic DNA, and DreamTaq Green PCR Master Mix (2 ×; Thermo Scientific, Waltham, MA). The primer sequences and PCR conditions are listed in Table 1.

**Table 1:** Primer sequence and conditions of polymerase chain reaction.

	Gene	Primer	Primer sequence (5’ to 3’)	PCR condition	Production size (bp)
*L. caulleryi*	*Cyt b*	Lcytb_F	TAGTTTCATGGATATGTGGT	95.0°C 120 s (95.0°C 30 s, 58.0°C 30 s, 72.0°C 60 s)^35 cycle^ 72.0°C 600 s	236
		Lcytb_R	ACTTTGTGATAAGAATAGTACT		
	*Cox 3*	Lcox3_F	TAACATTCTAGATGATGTTAA	95.0°C 120 s (95.0°C 30 s, 56.0°C 30 s, 72.0°C 60 s)^35 cycle^ 72.0°C 600 s	294
		Lcox3_R	GTAAAAGCACATTTAGTTTC		
*G. gullaus*	*Cyt b*	Gcytb_F	TAGTAGAGTGAGCCTGAGGG	95.0°C 120 s (95.0°C 30 s, 63.0°C 30 s, 72.0°C 60s)^35 cycle^ 72.0 °C 600 s	231
		Gcytb_R	GGCTAGTGTTAGGAATGGGGTG		
	*Cox 3*	Gcox3_F	AATCCTCCTAGCCTCAGGAGT	95.0°C 120 s (95.0°C 30 s, 54.0°C 30 s, 72.0°C 60 s)^35 cycle^ 72.0°C 600 s	325
		Gcox3_R	GTCAGATGATATCTACGAAG		

Because the sensitivity with which disease is detected is crucial for laying farms, we serially diluted the blood samples by adding infected blood to uninfected blood. The sensitivity tests were performed in pools ranging from 1 to 100.

### Quantification of Artemisinin Concentration in *A. annua* Through HPLC

Dry *A. annua* (Yufeng Biotechnology, China) was ground into powder. This powder was packed into colorless capsules that were then administered to the chickens as an oral additive.

Artemisinin concentrations in the *A. annua* (Yufeng Biotechnology) were quantified through HPLC. The materials used (aside from *A. annua*) are listed as follows: artemisinin 98% (Sigma, Burlington, VT), absolute ethanol 99.9% (Echo Chemical, Taichung, Taiwan), HPLC-grade methanol 99.9% (Duksa, Gyeonggido, South Korea), sodium hydroxide 99% (Sigma), acetic acid 99% (Sigma), sodium hydrogen phosphate (Acros Organi, Geel, Belgium), sodium dihydrogen phosphate (Echo Chemical), a 0.45-μm syringe filter (PureTech, Boston, MA), and a 20-mL syringe (Top Surgical, Kaohsiung, Taiwan).

HPLC was conducted using the HITACHI CM5000 HPLC system, which comprises a CM 5110 Pump, CM 5260 Auto Sampler with a 20-μL injection loop, CM 5310 Column Oven, and CM 5410 UV Detector (through which absorbance was recorded at 260 nm).

HPLC-ultraviolet was performed on an ACE Excel 5 C18-AR column (250 × 4.6 mm^2^, 5 μm) at 30°C. The mobile phases were 0.01 M sodium hydrogen phosphate and sodium dihydrogen phosphate buffer solution (water: methanol ratio = 55:45), and the flow rate was 1.0 mL/min (Diawara et al., 2011).

Standard solutions of artemisinin were prepared by dissolving 10 mg of artemisinin in 10 mL of 1.0 mg/mL ethanol. Various concentrations of artemisinin were prepared: 0.05, 0.1, 0.15, 0.2, and 0.25 mg/mL, each supplemented by adding ethanol to 1 mL samples. The solutions were then mixed with 4 mL of 0.2% sodium hydroxide solution and hydrolyzed in water at 45°C for 30 min. After the solutions had cooled to room temperature, 5 mL of 0.02 M acetic acid solution was added, and the solutions were passed through a 0.45-μm syringe filter to obtain the standard product.

Using 50 mL of methanol, 1 g samples of *A. annua* powder were ultrasonicated at room temperature for 60 min. The lost weight with made up with methanol, and the solvent was filtered. In an oven, 25 mL samples of filtrate were dried. Subsequently, 5 mL of ethanol was added to dissolve the residue, after which 1 mL aliquots were drawn and placed in a 10 mL measuring flask. Subsequently, 4 mL of 0.2% sodium hydroxide solution was added to the filtrate, which was then hydrolyzed in water at 45°C for 30 min. After the hydrolyzed filtrate had cooled to room temperature, 5 mL of 0.02 M acetic acid solution was added. The test product was obtained after the passage of the filtrate solution through a 0.45-μm syringe filter. All samples were subjected to 5 repeated tests.

### Animal Experimentation

#### Experimental Infection and Grouping

Sporozoite suspensions were inoculated into 43 two-wk-old male Lohmann chicks through the wing vein. The chicks were randomly divided into groups. The control group was given empty capsules. Each of the 2 experimental groups was divided into a full-period subgroup and a half-period subgroup. Specifically, half of the animals in each of the 2 experimental groups were given either 40 mg/kg sulfadimethoxine daily or 0.5 g of *A. annua* powder (containing 0.096 mg of artemisinin) daily from d 0 postinoculation. The other half was given either 40 mg/kg sulfadimethoxine daily or 0.5 g of *A. annua* powder (containing 0.096 mg of artemisinin) daily from day 14 postinoculation.

#### Measurement Items

Body weight and rectal temperature of chicks were recorded each week until day 35 postinoculation. Daily records were kept on mortality rate and on the pale comb rate and green feces rate. PCR detection of *L. caulleryi* was conducted each week. After the animal death, necropsy was performed. Furthermore, tissue sections were collected and subjected to hematoxylin and eosin staining and tissue PCR.

### Ethics Statement

All animal experiments were performed according to the guidelines established by the Institutional Animal Care and Use Committee (**IACUC**) of National Chung Hsing University. The study protocol was approved by the Ethics Committee of the National Chung Hsing University (IACUC No.: 107-042).

### Statistical Analysis

One-way analysis of variance was performed on the body weight and rectal temperature data using a general linear model. Among-group differences are presented as the least square mean. Data on the mortality, pale comb, green feces, and positive PCR rates were analyzed using the FREQ procedure with a chi-square test. All statistical analyses were conducted using SAS software (SAS, 2012)

## RESULTS

### Laboratory Colonization of *C. arakawae*

The *C. arakawae* samples were collected on 74 occasions. The results on the culture at different stages are presented in Table 2. The number of individuals captured on each occasion ranged widely (138–976) because of variations in wind, temperature, and humidity. An average of 3,224 eggs was laid by female midges in each collection. The hatching rate, pupation rate, emergence rate, and blood-sucking rate were 14.8%, 82.4%, 88.0%, and 84.2%, respectively. The egg, larval, pupal, and imago stages lasted 2 to 3, 10 to 14, 2 to 3, and 4 to 6 day, respectively. A complete life cycle of *C. arakawae* lasted approximately 21 to 25 day. The successful subculture of *C. arakawae* in this experiment enabled the establishment of an animal model of leucocytozoonosis.

**Table 2:** The number of *C. arakawae* at different stages reared in the laboratory from 74 captures.

Items	Range	Average	Duration, Date
Captured number	138∼976	502	0
Survival number of females	37∼342	173	2
Number of eggs	650∼6,830	3224	3
Number of larvae	59∼1278	476	11
Pupation rate, %	75.5∼92.8	82.4 (392/476)	15
Emergence rate, %	85.4∼97.9	88.0 (345/392)	19
Blood-sucking rate, %	76.5∼88.1	84.2 (139/165, female only)	21

### Experimental Infection of Chickens With *L. caulleryi*

**Figure 1:**
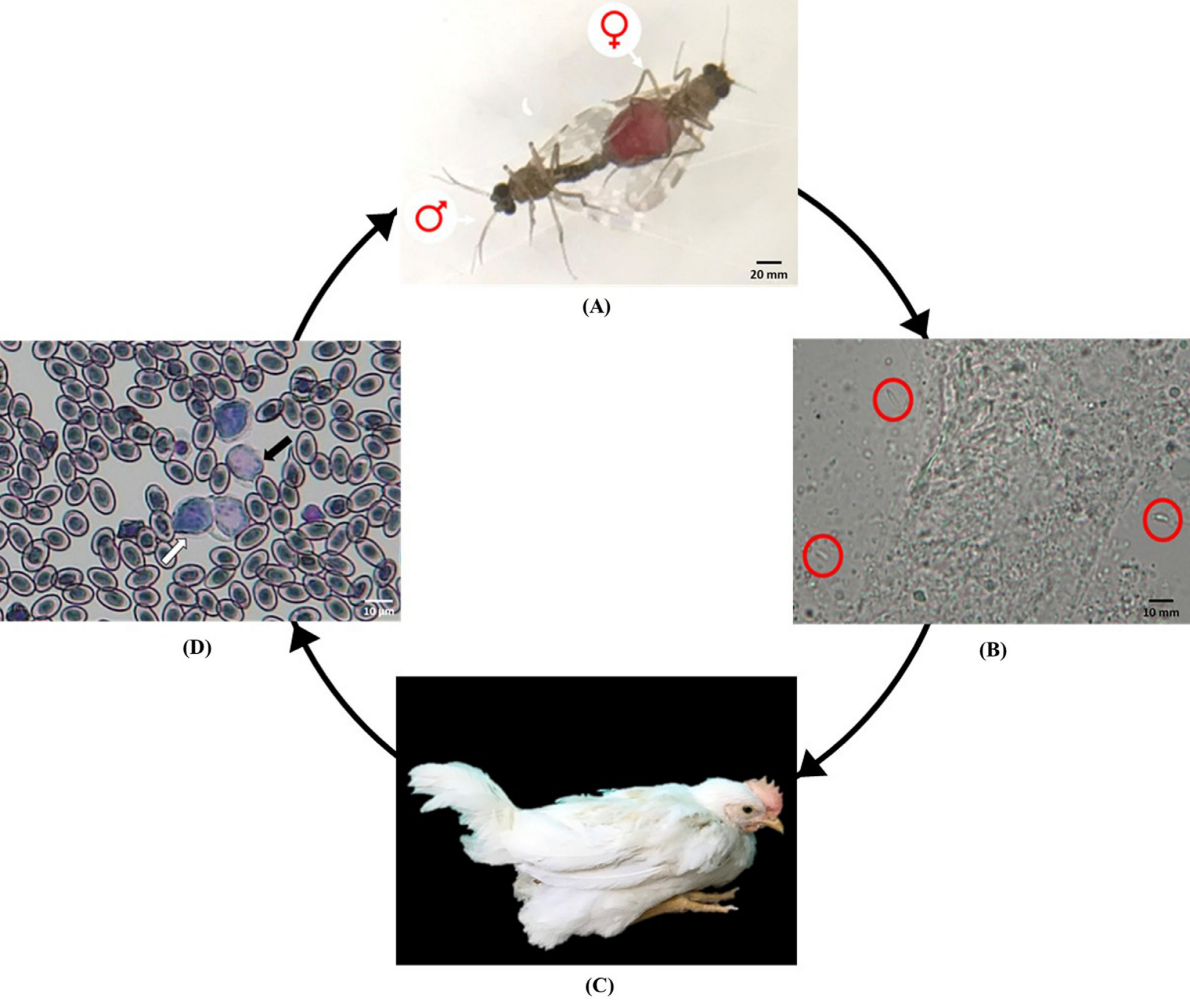
Life cycle of Leucocytozoonosis. (A) Observation of the copulation of wild *C. arakawae* and the female 2 days after blood sucking. (B) Anatomy of salivary glands of female *C. arakawae.* Red circle showed the sporozoites. (C) The Lohmann roosters showed clinical symptoms with of depression, pale cockscomb and green diarrhea after infection. (D) Gametophyte of *Leucocytozoon caulleryi* can be observed by blood smear on the 21st day after artificial infection. Solid arrow and hollow arrow are male and female gametophyte; Nucleated red blood cells scattered around.

Figure 1 showed the life cycle of leucocytozoonosis. As mentioned, the female midges were forced to suck the blood of sick chickens, after which they were reared in a chamber where the temperature and humidity were maintained at 25 ± 2°C and 90 ± 3%. After 54 hours had elapsed, we confirmed that the sporozoites had formed in the salivary glands of the female midges; we then removed these glands with microneedles. Transparent sickle-shaped spores gushed out, and the activity state was fast swinging left and right (Figure 1B)

### Diagnostic Methods

#### Blood Smears

As shown in Figure 1D, gametocytes had developed to the third to fourth stage on by day 18 to 19 postinoculation. Through Giemsa staining, pale pink male gametes were differentiated from the blue-violet female gametes. Parasite concentrations in the blood peaked on day 21 to 22 postinoculation. The ratio of female to male gametes was approximately 2:1. On day 26 to 27 postinoculation, the gametes in the blood smears had almost disappeared. Moreover, the number of white blood cells in the chickens had increased. A notable increase in the number of monocytes and macrophages was observed.

#### Molecular Diagnosis

**Figure 2:**
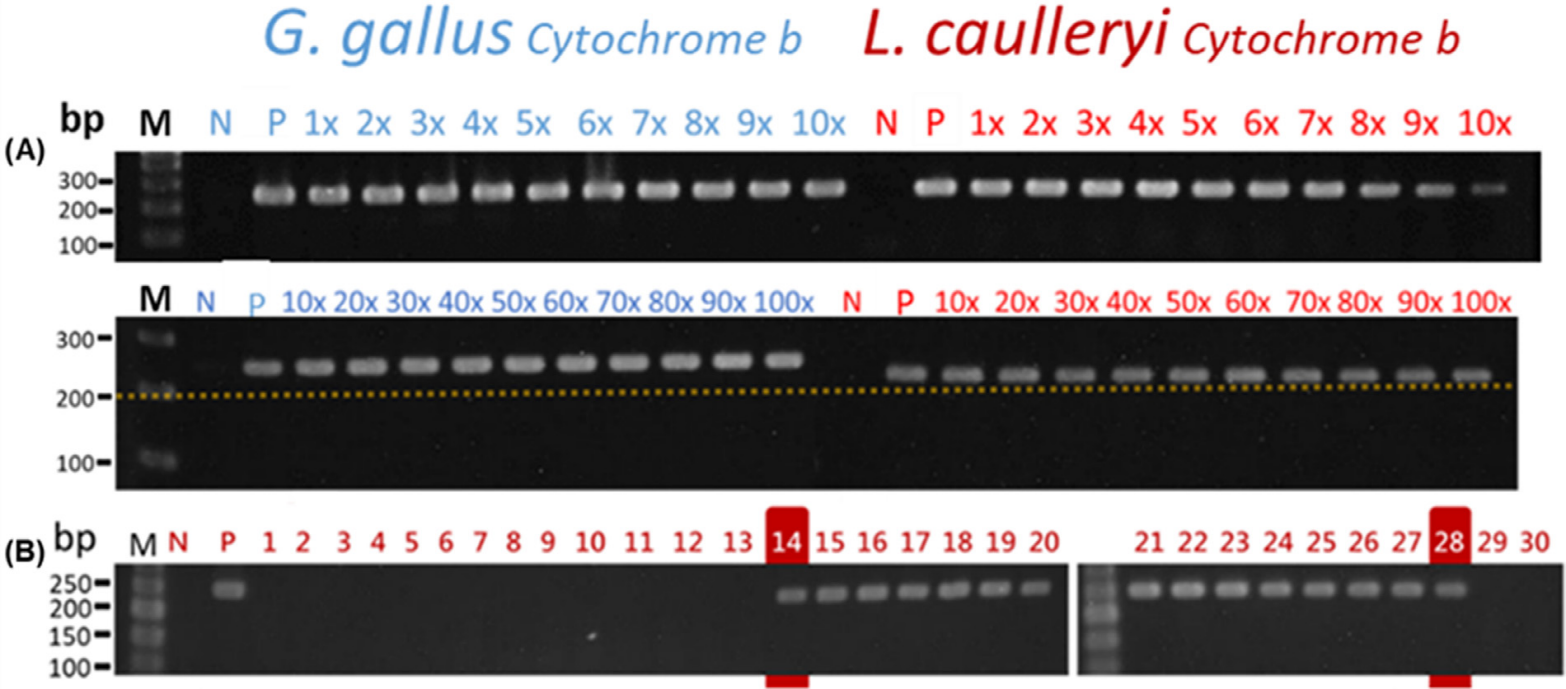
Detection of *L. caulleryi* in the blood by agarose gel electrophoresis of PCR products. (A) Minimum detectable concentration of protozoa in the blood. (B) Difference day after artificially infected. M: DNA ladder (100 and 50 bp); Number in front of X indicates the dilution factor; P, positive control; N, negative control.

Through the use of PCR, the *cytochrome b* gene of *L. caulleryi* in the infected chickens was detected. Agarose gel electrophoresis indicated that the fragment of the *cytochrome b* gene had approximately 236 base pairs, indicating the presence of *L. caulleryi* (Figure 2A). The infection rate was 100% on day 14 postinoculation. The PCR results changed from positive to negative until day 29 postinoculation. Except for the blood tests, the tests of the pancreas, liver, lungs, pancreas, spleen, heart, pectoral muscles, and leg muscles also yielded positive results. According to the results of the sensitivity test, *L. caulleryi* was present in 1 infected chicken out of a pool of 100 individuals (of which 99 were healthy) was detectable by PCR (Figure 2A).

### Quantification of Artemisinin Concentration in Artemisia annua Through HPLC

**Figure 3:**
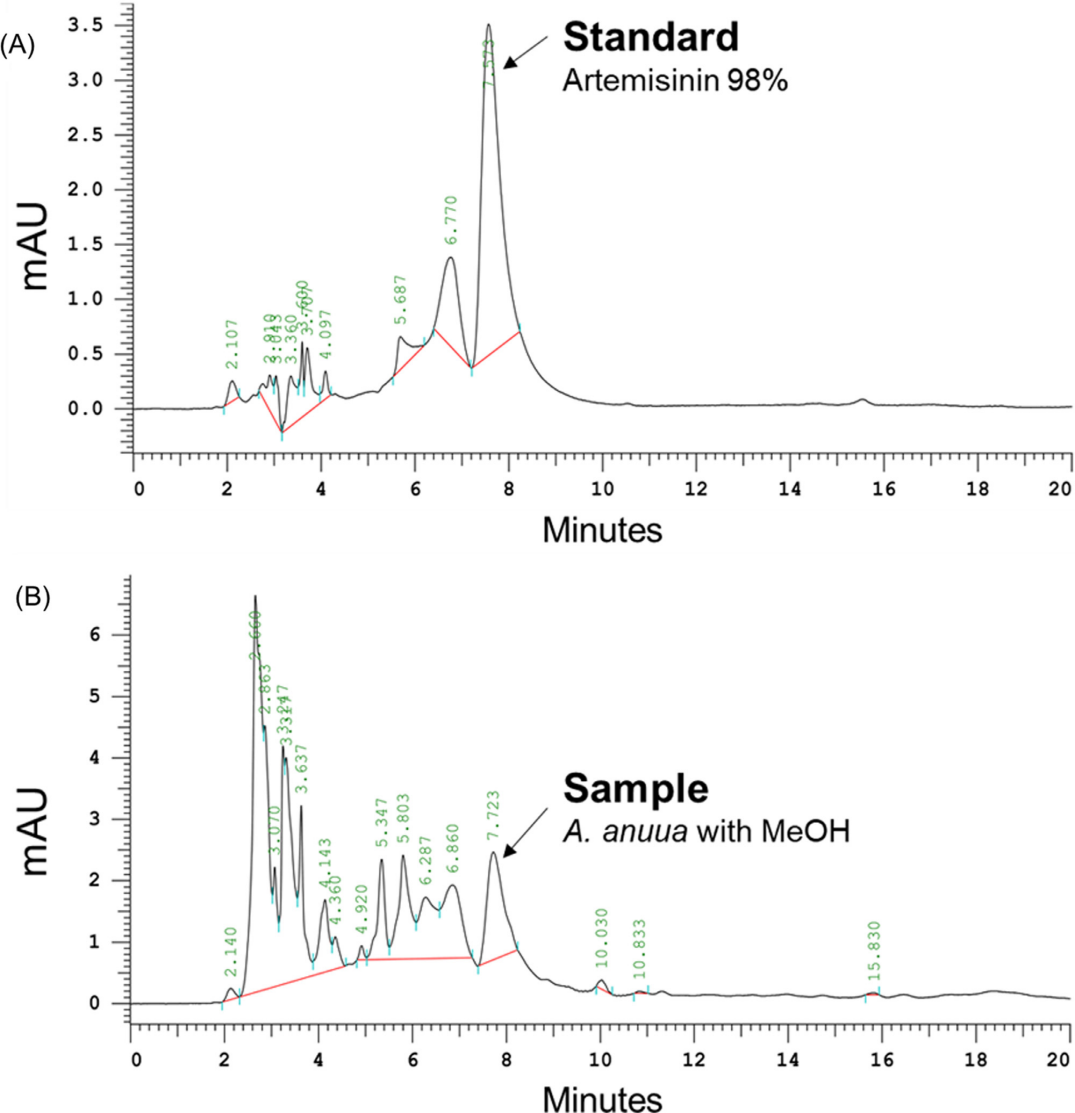
HPLC chromatogram of *A. annua*. (A) 98% Artemisinin standard. (B) Sample 1 g *A. annua* powder with methanol.

As mentioned, standards at concentrations of 0.05 to 0.25 μg/mL (0.05, 0.10, 0.15, 0.20, and 0.25) were freshly prepared and used in the HPLC. The identification and quantification of artemisinin were conducted through a comparison of its retention time in the peak area found in the plant sample with the artemisinin peak from a standard solution that was injected (Figure 3). In the HPLC-ultraviolet (absorbance at 260 nm) test, derivatized artemisinin was eluted at 7 min. The linear regression line was y = 369,600 x – 13,670. The correlation coefficient r² in the linear plot of the standard curve generated was 0.9923. The artemisinin concentration in the *A. annua* powder was 0.192 ± 0.061 mg/g.

### Animal Experimentation

#### Body Weight Gain and Rectal Temperature

The results on body weight gain from day 0 to 35 postinoculation are presented in Table 3. No significant differences in body weight gain were observed among the groups before day 14 (*P* > 0.05). However, the body weight gain of the control group was significantly lower than those of the experimental groups from day 14 to 28 (*P* < 0.05). In addition, the body weight gain of the antibiotic and *A. annua*. groups receiving full- and half-period treatment were not significantly different (*P* > 0.05). On day 14, the average rectal temperature was 43.0°C (0.82°C increase from that on d 0), with no significant differences among the groups. After day 14, rectal temperature decreased; specifically, the rectal temperature in both the antibiotic and *A. annua* groups decreased significantly compared with that in the control group (Table 3).

**Table 3:** Mean ± SE of body weight gain and in different periods among five groups.

Different periods	Control	*Sulfadimethoxine*		*A. annua*		*P* value
		Full period	Half period	Full period	Half period	
Body weight gain						
D 0 (initial body weight)	192.9 ± 7.8	193.6 ± 5.1	194.9 ± 8.1	188.9 ± 3.4	179.6 ± 2.7	> 0.1
D 0 to D 14	133.8 ± 15.4	136.0 ± 8.8	144.1 ± 14.8	143.4 ± 9.7	154.3 ± 10.5	> 0.1
D 0 to D 21	208.8 ± 18.6	235.6 ± 11.2	234.4 ± 19.0	219.0 ± 15.2	260.4 ± 13.4	> 0.1
D 0 to D 28	316.9 ± 24.3	371.2 ± 14.0	348.6 ± 26.1	335.5 ± 12.2	391.0 ± 15.3	0.08
D 0 to D 35	436.4 ± 24.8	515.9 ± 18.1	488.7 ± 31.6	490.9 ± 19.3	523.3 ± 12.2	0.08
D 14 to D 21	75.0 ± 6.1^c^	99.6 ± 8.8^ab^	90.3 ± 8.0^ab^	75.6 ± 10.9^bc^	106.1 ± 7.6^a^	0.04
D 14 to D 28	183.1 ± 11.6^b^	235.3 ± 12.8^a^	204.6 ± 17.6^ab^	191.9 ± 11.2^b^	236.7 ± 7.9^a^	0.01
D 14 to D 35	294.5 ± 17.8^b^	379.9 ± 19.1^a^	344.7 ± 26.9^ab^	347.5 ± 16.2^ab^	369.0 ± 6.5^a^	0.03
Change of rectal temperature						
D 0 to D 14	0.67 ± 0.14	0.71 ± 0.20	0.91 ± 0.10	0.50 ± 0.17	0.90 ± 0.13	> 0.1
D 14 to D 21	−0.29 ± 0.12^bc^	−0.20 ± 0.10^c^	−0.53 ± 0.06^ab^	−0.10 ± 0.14^c^	−0.77 ± 0.07^a^	0.01
D 14 to D 28	−0.11 ± 0.17^b^	−0.39 ± 0.16^ab^	−0.58 ± 0.06^a^	−0.14 ± 0.09^b^	−0.53 ± 0.06^a^	0.01
D 14 to D 35	−0.16 ± 0.11^b^	−0.38 ± 0.14^ab^	−0.43 ± 0.04^a^	−0.06 ± 0.09^b^	−0.43 ± 0.02^a^	0.01

a-c Mean ± standard error within the same row with different superscript letter are significantly different (*P* < 0.05).

#### Clinical Symptoms

In general, the infected animals exhibited obvious symptoms on day 13 to 14 postinoculation. Mortality did not significantly differ among the groups. On day 15, one chick in the control group died. All the infected animals exhibited clinical symptoms, including depression, pale comb, and green feces, on day 14. Lower rates of pale comb and green feces were observed in the chicks in the experimental groups receiving full-period treatment. The experimental groups had a higher recovery rate than compared with the control group, and they recovered 2 to 3 days earlier than did the control group. By day 31 after experimental infection, all the animals had recovered.

#### Necropsy, Tissue Section, and Hematoxylin and Eosin Stain

**Figure 4:**
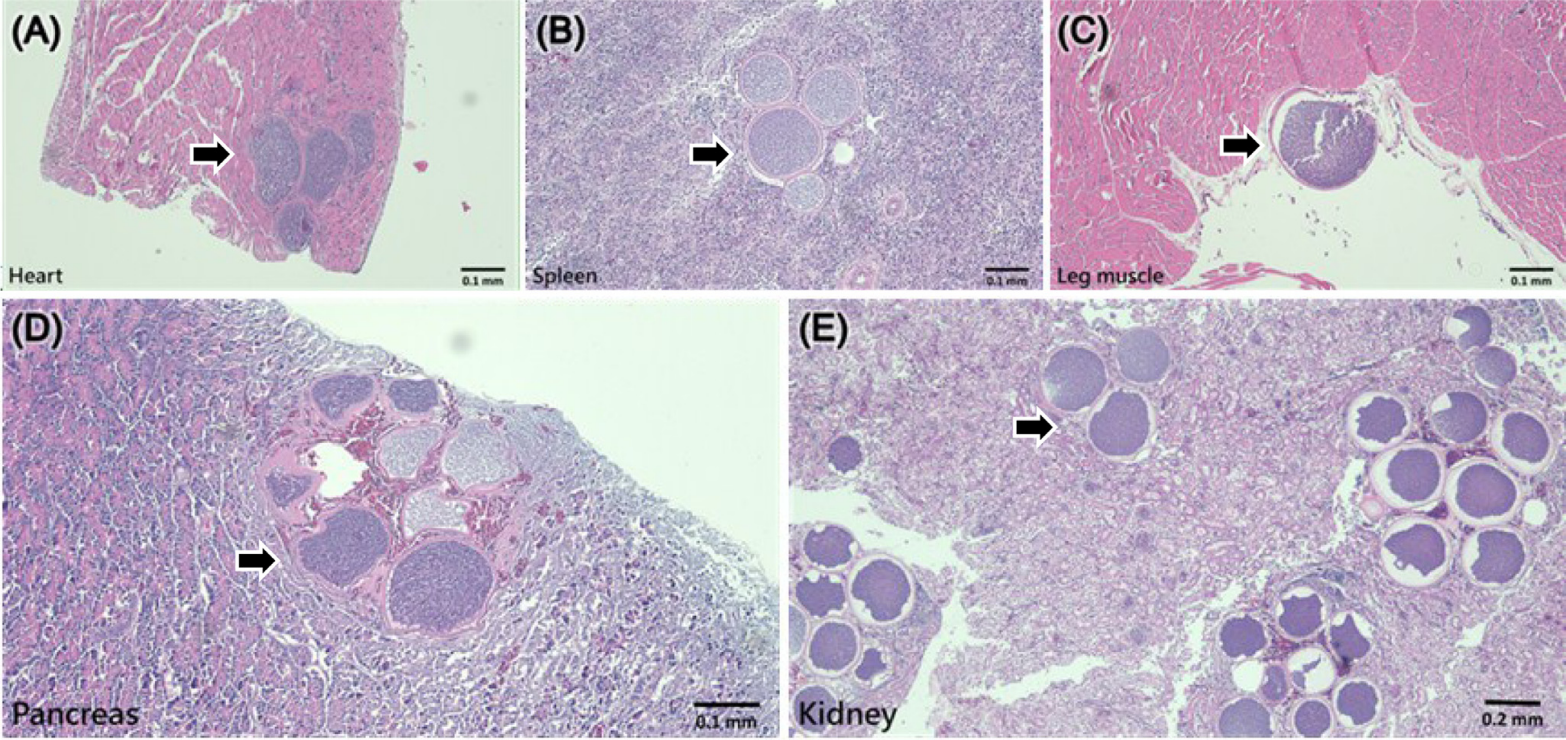
Tissue section on d 14 after infected with *L. caulleryi*. (A) Heart, (B) spleen, (C) leg muscle, (D) pancreas and (E) kidney. Arrow is second generation schizont of *L. caulleryi*.

A necropsy of the infected chicks revealed white pinpoint spots and blood spots caused by the development of second-generation schizonts in the organs, including the pancreas, liver, lungs, and digestive system (mesentery and intestinal muscle layer), pectoral muscles, and epicardial membrane. Hematoxylin and eosin staining of tissue sections indicated that the parasites were mostly concentrated in the lungs, kidneys, and pancreas, causing severe coagulative necrosis, multifocal hemorrhage, and pleomorphic lymphocyte infiltration. Furthermore, eosinophilic amorphous residues were observed in the schizont capsules of *L. caulleryi* (Figure 4).

## DISCUSSION

Leucocytozoonosis in chickens is a vector-borne disease. *C. arakawae*, the intermediate host of *L. caulleryi*, is a vector that causes the spread of leucocytozoonosis in tropical countries. With regard to the prevention of vector-borne disease, the reduction of vector populations, such as that through the use of insecticides or mosquito traps during the epidemic season, is the first step (Chiu, 2010; White et al., 2014). To perform studies on leucocytozoonosis, the successful laboratory colonization of *C. arakawae* is essential. Methods for colonization of *C. arakawae* are readily available (Morii and Kitaoka, 1968b); however, the present method can be easily followed even by less trained persons. To obtain infectious sporozoites from *C. arakawae* (Morii and Kitaoka, 1968a) conducted experiments on the effects of temperature on sporogony and on sporozoite susceptibility. When the temperature was 20°C, 25°C, and 30°C, sporozoites appeared in the salivary glands of the midges 4, 3, and 2 days after they fed on blood. In the present study, the midges were reared at 25 ± 2°C and under a relative humidity of 90 ± 3%, and the infectious sporozoites to which the chicks were susceptible were detected 54 h after the midges sucked the animals’ blood.

According to Akiba (1970), stage II protozoans can be detected in smear preparations of peripheral blood stained with Giemsa solution at the earliest on day 14 to 15 after inoculation with sporozoites. The present results demonstrate that it is highly feasible to diagnose leucocytozoonosis using PCR to detect mitochondrial gene fragments in *L. caulleryi* in the blood of sick chickens. Moreover, PCR detection allows for earlier diagnosis than do conventional methods on day 14 after experimental infection in this case. Until day 29 postinoculation, the PCR results changed from positive to negative. PCR was also determined to have high sensitivity and high accuracy. Detection using pooled samples is a quick and effective strategy for diagnosing disease in epidemiology. Regarding the PCR detection allows for earlier diagnosis than do conventional methods on day 14 after experimental infection in this case. Performance of chickens show potential clinical symptoms can be observed on laying farms, after which individual animals can be pooled for rapid diagnosis.

Artemisinin, an active ingredient in *A. annua*, has been developed as an antimalarial and is used in artemisinin-based combination therapies. Artemisinin mainly activates the antiprotozoal mechanism through free iron ions produced from the decomposition of hemoglobin (Wang et al., 2015). This mechanism depolarizes the cell membrane (Wang et al., 2010) and destroys the protein structure of the protozoan (Antoine et al., 2014), inhibits protozoan entrapment (Hoppe et al., 2004), and leads to loss of activity of the protozoan sarco/endoplasmic reticulum Ca^2+^-ATPase orthologue (PfATP6) enzyme by the structure endoperoxide bridge of artemisinin (Eckstein-Ludwig et al., 2003).

In the present study, we assessed the effects of *A. annua* powder on leucocytozoonosis in chickens. Body weight gain and rectal temperature significantly increased from day 14 to 35 postinoculation in both the antibiotic and *A. annua* groups, but significant differences were noted between them. Body weight gain did not differ significantly between the full and half periods, possibly because the schizonts settled themselves in the endothelial cells of blood vessels and in organs with a distinct capsule during the first 14 day. Akiba et al. (1968) investigated the process of schizont development in experimentally infected chickens. The cycle began with the inoculation of sporozoites into the wing vein, which then invaded to the endothelial cells of blood vessels, where they formed schizonts. The schizonts were released from host cells 9 day postinoculation, transported by the blood to various organs and tissues where they developed into independent schizonts. On day 14, the organ schizonts, which were being released into the bloodstream by thousands of merozoites that invaded the red blood cells, began to enter the gametes. Gametogony of the protozoans was observable in smear preparations of peripheral blood stained with Giemsa solution from day 14, the day marking the onset of the disease and the clear presentation of clinical symptoms (Akiba, 1960). Drug administration could not mitigate weight loss before disease onset. By contrast, drug administration after disease onset significantly increased weight gain. Furthermore, the concentration of gametocytes in the *A. annua* group was lower than that in control group. These indicate that artemisinin mainly acted on the gametogony of the *L. caulleryi*.

The increase in body temperature was caused by activation of the animals’ immune response by the protozoan. A study demonstrated that chickens infected with protozoans had an increased secretion of the proinflammatory cytokine interleukin 6 (Lynagh et al., 2000), which not only mediates the migration of immune cells to the pathogen but also affects the activity of the hypothalamic thermoregulation center, causing fever (Kozak et al., 1998). Williams (2005) was the first to report that the core temperatures of chickens infected with avian malaria exceeded 42.0°C. In the present study, from day 0 to 14 (before disease onset), the rectal temperature of the experimental groups, including the full-period subgroups, gradually increased. From day 14 to 35, the rectal temperature of the chickens receiving *A. annua* considerably and sharply decreased.

To prevent and control leucocytozoonosis, there are 2 strategies for farmers to use *A. annua* powder in the field or farm. First, they can detect *L. caulleryi* in pooled blood by highly sensitive PCR. If the PCR result is positive, they can start to feed the *A. annua* powder for 2 to 4 weeks since the earlier stage of leukocytozoonosis. The second strategy is directly feeding *A. annua* powder without PCR detection. Because the leukocytozoonosis usually occurs during March to April and October to November in Taiwan, we suggest that farmers can feed the *A. annua* powder on February and September each year to prevent the disease or decrease the morbidity rate.

## CONCLUSIONS

The successful subculture of *C. arakawae* was the foundation of the experimental infection model. PCR was determined to have high sensitivity and accuracy in *L. caulleryi* detection; this facilitated early diagnosis and treatment. Furthermore, the daily administration of 0.5 g of *A. annua* powder to the chickens not only increased body weight gain but also reduced the parasite concentration of *L. caulleryi*, which in turn reduced mortality and the occurrence of pale comb and green feces. The findings serve as a reference for further research on leucocytozoonosis in chickens.
